# Thymic Egress Is Regulated by T Cell-Derived LTβR Signal and *via* Distinct Thymic Portal Endothelial Cells

**DOI:** 10.3389/fimmu.2021.707404

**Published:** 2021-07-01

**Authors:** Huan Xia, Suijuan Zhong, Yixiao Zhao, Boyang Ren, Zhongnan Wang, Yaoyao Shi, Qian Chai, Xiaoqun Wang, Mingzhao Zhu

**Affiliations:** ^1^ Key Laboratory of Infection and Immunity, Institute of Biophysics, Chinese Academy of Sciences, Beijing, China; ^2^ College of Life Sciences, University of the Chinese Academy of Sciences, Beijing, China; ^3^ State Key Laboratory of Cognitive Neuroscience and Learning, Institute of Biophysics, Chinese Academy of Sciences, Beijing, China

**Keywords:** thymus, thymocyte, cell migration, endothelial cell, lymphotoxin beta receptor

## Abstract

Thymic blood vessels at the perivascular space (PVS) are the critical site for both homing of hematopoietic progenitor cells (HPCs) and egress of mature thymocytes. It has been intriguing how different opposite migrations can happen in the same place. A subset of specialized thymic portal endothelial cells (TPECs) associated with PVS has been identified to function as the entry site for HPCs. However, the cellular basis and mechanism underlying egress of mature thymocytes has not been well defined. In this study, using various conventional and conditional gene-deficient mouse models, we first confirmed the role of endothelial lymphotoxin beta receptor (LTβR) for thymic egress and ruled out the role of LTβR from epithelial cells or dendritic cells. In addition, we found that T cell-derived ligands lymphotoxin (LT) and LIGHT are required for thymic egress, suggesting a crosstalk between T cells and endothelial cells (ECs) for thymic egress control. Furthermore, immunofluorescence staining analysis interestingly showed that TPECs are also the exit site for mature thymocytes. Single-cell transcriptomic analysis of thymic endothelial cells suggested that TPECs are heterogeneous and can be further divided into two subsets depending on BST-1 expression level. Importantly, BST-1^hi^ population is associated with thymic egressing thymocytes while BST-1^lo/−^ population is associated with HPC settling. Thus, we have defined a LT/LIGHT-LTβR signaling–mediated cellular crosstalk regulating thymic egress and uncovered distinct subsets of TPECs controlling thymic homing and egress, respectively.

**Graphical Abstract:**
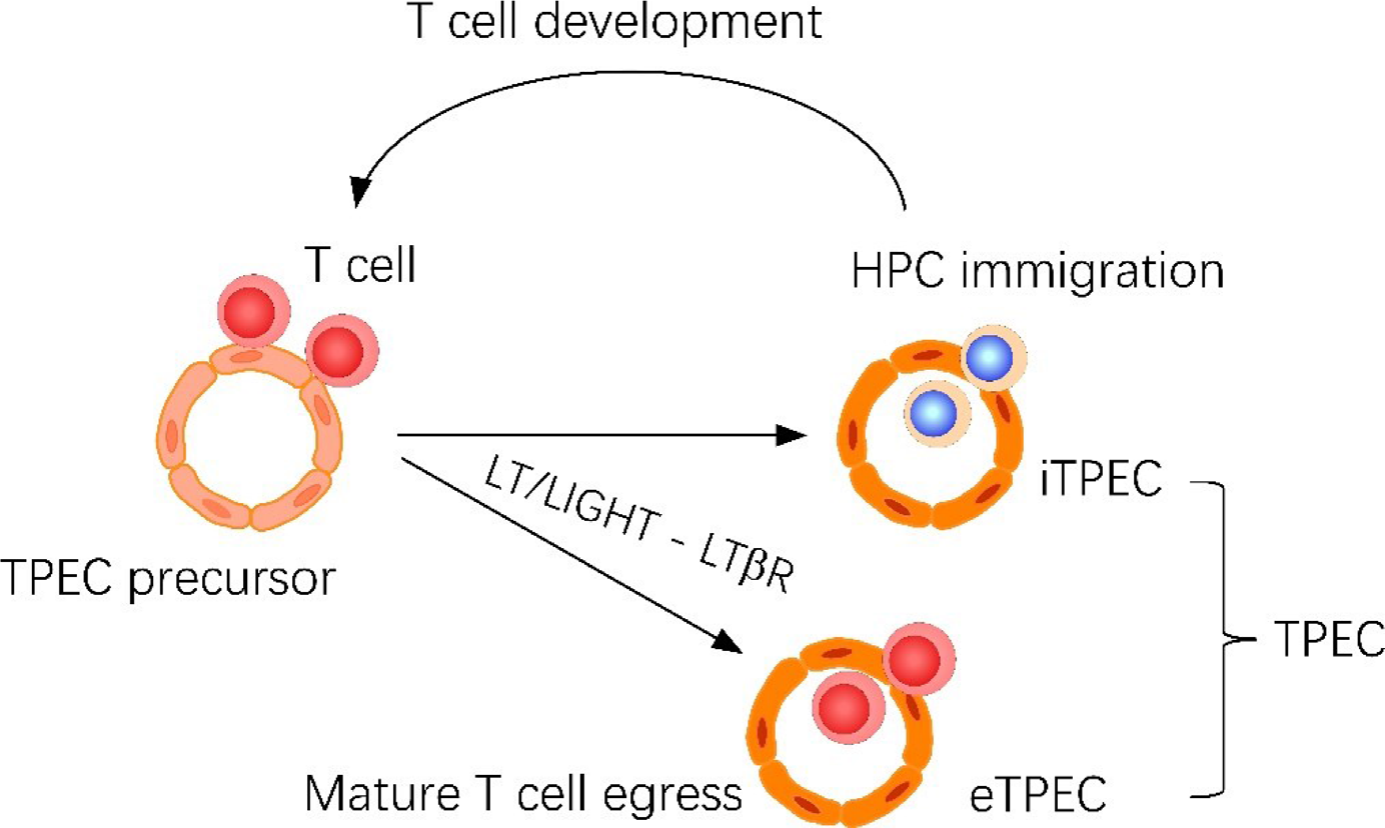
Thymic portal endothelial cells (TPECs) are associated with both thymic homing of hematopoietic progenitor cells (HPCs) and thymic egress of mature thymocytes. Current study suggests that TPECs are actually composed of two subsets, iTPECs and eTPECs, associated with HPC immigration and mature thymocyte egress, respectively. •T cell derived LT/LIGHT ligands regulates thymic egress of mature thymocytes. •Endothelial LTβR signaling regulates thymic egress of mature thymocytes. •scRNA-seq reveals the heterogeneity of thymic portal endothelial cells. •Thymic egress of mature thymocytes and homing of HPCs take place at different thymic portal endothelial cell populations.

## Introduction

T cell development in the thymus starts with hematopoietic progenitor cells (HPCs) transendothelial migration into thymic parenchyma and ends with mature thymocytes reverse-transendothelial migration to peripheral blood (1–3). Mori et al. found that the perivascular space (PVS) located around vessels in the medulla and at the cortico-medullary junction (CMJ) contains HPCs and mature T cells, but not immature thymocytes (4), suggesting PVS is a transit pathway for progenitor cells to immigrate into the thymus and for mature T cells to emigrate from the thymus. Indeed, upon adoptive transfer, CD117-positive bone marrow progenitor cells have been found to be settled within PVS. Zachariah et al. used an intravascular procedure to directly label emigrating cells and found that mature thymocytes exit also *via* blood vessels at the CMJ (5). These previous studies make it obscure how both thymic entry and egress take place at the same place. Our previous work has identified and characterized thymic portal endothelial cells (TPECs) as the cellular basis for thymic homing of HPCs (6). However, it remains unclear which type of thymic endothelial cells (ECs) is responsible for thymic egress and what is the relationship between that and TPECs.

The lymphotoxin beta receptor (LTβR) signaling pathway, engaged by the ligands of lymphotoxin (LT) and LIGHT, has crucial roles in the homeostatic maintenance and function of specialized vascular ECs, which play important roles regulating lymphoid tissue–associated cell migration. In lymph nodes (LNs), dendritic cells (DCs) provide LT to control the differentiation and function of high endothelial cells (HECs), which are vascular ECs specialized for lymphocytes homing (7). In the thymus, positively selected T cells, but not DCs or B cells, control TPECs *via* LT/LIGHT-coordinated signals during HPC homing (6). Recently, James et al. showed that the requirement of LTβR in thymocyte emigration is distinct from its control of thymic epithelium and instead maps to expression by endothelial cells (8). Likewise, they observed significant loss of TPECs in mice with LTβR loss on endothelium and suggested that TPECs are required for thymic egress. However, it remains unclear which LTβR ligand and which type of cells deliver the ligand signal to orchestrate thymic emigration *via* thymic ECs. Importantly, how TPECs coordinate both thymic homing and egress is intriguing.

Here, we found that positively selected T cells deliver LT and LIGHT signals to endothelial LTβR for thymic emigration control. Interestingly, two subsets of TPECs were identified by single-cell RNA sequencing (scRNA-seq), with preferential association with thymic settling HPCs and egressing mature thymocytes, respectively. Thus, our data suggested that thymic HPC homing and mature thymocytes egress actually occur at different subsets of TPECs, both of which are controlled by LTβR signaling.

## Materials and Methods

### Mice

Wild-type C57BL/6 mice were purchased from Vital River, a Charles River company in China. *Tek* ^Cre^ mice were obtained from Nanjing Biomedical Research Institute. *Tcra* ^−/−^, *K14* ^Cre^, and CD45.1 mice were obtained from The Jackson Laboratory. *Ltbr* ^−/−^, *Ltbr* ^fl/fl^, and *Light* ^−/−^ mice were as previously described (6). Rag2pGFP mice were provided by Qing Ge (Peking University School of Basic Medical Sciences). *Cd11c* ^Cre^ mice were provided by Baidong Hou (Institute of Biophysics, Chinese Academy of Sciences). *Lta* ^−/−^ mice were provided by Burkhard Ludewig (Kantonal Hospital, Switzerland). *Bst1* ^−/−^ mice were generated by Cyagen Biosciences company. CRISPR/Cas9 technique was used to deplete exons 5 and 6 of *Bst1*. Four- to 6-week-old sex-matched mice were used unless described otherwise. All mice were on the C57BL/6 background and were maintained under specific pathogen-free conditions with approval by the institutional committee of the Institute of Biophysics, Chinese Academy of Sciences.

### Isolation of Thymic ECs

Thymus tissues were digested with 0.2 mg/ml collagenase I (Sigma), 1 U/ml dispase I (Corning), and 0.06 mg/ml DNase I (Roche) in RPMI 1640 medium with 2% fetal bovine serum (FBS) for 1 h at 37°C. The digestion was washed with cold PBS and filtered through a 70-μm cell strainer (Biologix Group). The stromal cells were enriched by discontinuous density gradient centrifugation in Percoll (GE Healthcare) (bottom layer = 1.115 g/ml; middle layer =1.06 g/ml; top layer = 2% FBS RPMI 1640). The cells recovered from the upper interface were washed and stained with antibodies for flow cytometric analysis or cell sorting.

### Intravascular Thymocyte Labeling

This method has been previously described (5). Briefly, 1 μg of PE-conjugated rat anti-mouse CD4 antibody (GK1.5) (eBioscience) was intravenously injected, and mice were sacrificed 3–5 min later. For flow cytometry, mice were sacrificed and thymi were dissociated in ice-cold PBS containing 2% FBS immediately after harvest. For immunofluorescence and confocal microscopy, thymi were fixed in IC Fixation Buffer (eBioscience, 00-8222-49) and were shaken at 4°C, overnight. Thymi were dehydrated in 30% sucrose until they sank to the bottom at 4°C and then were embedded in OCT (Sakura Finetek), frozen, and stored at −80°C.

### Immunofluorescence Microscopy

Thymus tissues were freshly embedded in OCT compound and snap frozen in liquid nitrogen. Cryosections that were 6 μm thick were air-dried and fixed for 10 min in cold acetone. For fixation by IC buffer or paraformaldehyde, tissues were first fixed and then dehydrated before embedded in OCT compound. Cryosections were air-dried and used directly. Cryosections were blocked for 1 h in PBS containing 2% FBS and 1 mg/ml anti-FcγRII/FcγRIII (2.4G2) (in-house production). Cryosections were incubated overnight at 4°C with the following antibodies: anti-CD31 (MEC13.3) (eBioscience), anti-Ly6C (HK1.4) (BioLegend), anti-collagen IV (LSL), anti-GFP, anti-BST-1 (BP-3) (BioLegend), and anti-CD45.2 (104) (eBioscience). Unconjugated antibodies were detected with the following secondary antibodies: AlexaFluor®549-conjugated anti-rabbit IgG (Jackson) and TRITC-conjugated streptavidin (Jackson). Microscopic analysis was performed using a confocal microscope (Zeiss LSM-710), and the images were processed with ZEN 2010 software (Carl Zeiss, Inc.).

### Flow Cytometry and Cell Sorting

All antibodies used for flow cytometry were from BD Biosciences, eBioscience, or BioLegend. Flow cytometry data were acquired on an LSRFortessa (BD) with FACSDiva software (BD), and FlowJo software (TreeStar) was used for further analysis. Cell sorting was performed on a FACSAriaII or FACSAriaIII (BD). For mature thymocyte analysis, single-cell suspensions were stained with anti-CD4 (GK1.5), anti-CD8 (53-6.7), anti-CD62L (MEL-14), anti-CD24 (M1/69), anti-CD69 (H1.2F3), and anti-TCRβ (H57-597). For intravascular thymocyte labeling analysis, single-cell suspensions were stained with anti-CD8 and stained for anti-CD62L and anti-CD44 when the mice were not on Rag2pGFP background. For thymic endothelial cell staining, the samples were stained with antibodies against CD45 (30-F11), CD31 (MEC13.3), EpCAM (G8.8), P-selectin (RB40.34), BST-1 (BP-3), and Ly6C (HK1.4). Dead cells were excluded by staining with DAPI (Sigma).

### Bone Marrow Chimeras and HPC Homing Location Assay

For the bone marrow chimeras, 5 × 10^6^ bone marrow cells from donor mice were injected intravenously into congenic C57BL/6 host mice that had been lethally irradiated (1,000 rad). The chimeras were given prophylactic water containing antibiotics for 4 weeks following bone marrow transfer. The chimeras were analyzed 6–8 weeks after transplantation. To visualize the thymic seeding progenitor cells, CD45.2^+^Lin^−^c-Kit^+^ bone marrow progenitor cells were injected intravenously into the CD45.1^+^ recipients (0.5–2 × 10^6^ cells/mouse). Twenty hours later, the recipient mice were euthanized and the thymi were removed and frozen in OCT for immunofluorescence staining.

### Single-Cell RNA Sequencing

Cell suspensions were processed for single-cell RNA sequencing using Chromium Single Cell 3’ Library and Gel Bead Kit v2 (10X Genomics, PN-120237) according to 10X Genomics guidelines. Custom written scripts in Cellranger, R, R package: Seurat. Cell ranger 2.0.1 (http://10xgenomics.com) performed quality control and read counting of Ensemble genes with default parameter (v2.0.1) by mapping to mm10 mouse genome. We excluded poor quality cells after the gene-cell data matrix was generated by cell ranger software using the Seurat package (v2.3.4). Only cells that expressed more than 500 genes and less than 8,000 genes were considered, and only genes expressed in at least five single cells (0.1% of the raw data) were included for further analysis. Cells with the mitochondrial gene percentages over 15% were discarded. The data were normalized to a total of 1e4 molecules per cell for the sequencing depth by using Seurat package. Seurat package was used to perform linear dimensional reduction. Nine hundred ten highly variable genes with average expression between 0.0125 and 8 and dispersion greater than 2 were selected as input for principal component analysis (PCA). Then we determined significant PCs based on JackStrawPlot function. Strongly PC1-10 were used for t-Distributed Stochastic Neighbor Embedding (tSNE) to cluster the cells by FindClusters function with resolution 0.6. Clusters were identified by the expression of known cell-type markers. The differentially expressed genes (DEGs) of each cluster were identified by FindAllMarkers function (thresh.use = 0.25, test.use = “wilcox”). Wilcoxon rank sum test (default) and genes with average expression difference >0.5 natural log with P < 0.05 were selected as marker genes. The scRNA-seq dataset was deposited in NCBI gene expression omnibus, with an accession number of GSE174732 at the following link: https://www.ncbi.nlm.nih.gov/geo/query/acc.cgi?acc=GSE174732.

### Statistical Analysis

The statistical significance of the differences between sets of data was assessed by a two-tailed unpaired Student’s *t*-test unless stated otherwise. The results are expressed as the mean ± SEM. Differences with a P-value <0.05 are marked with asterisks. *P < 0.05; **P < 0.01; ***P < 0.001; ****P < 0.0001; n.s., not significant.

## Results

### LTβR Regulates Thymic Egress

**Figure 1:**
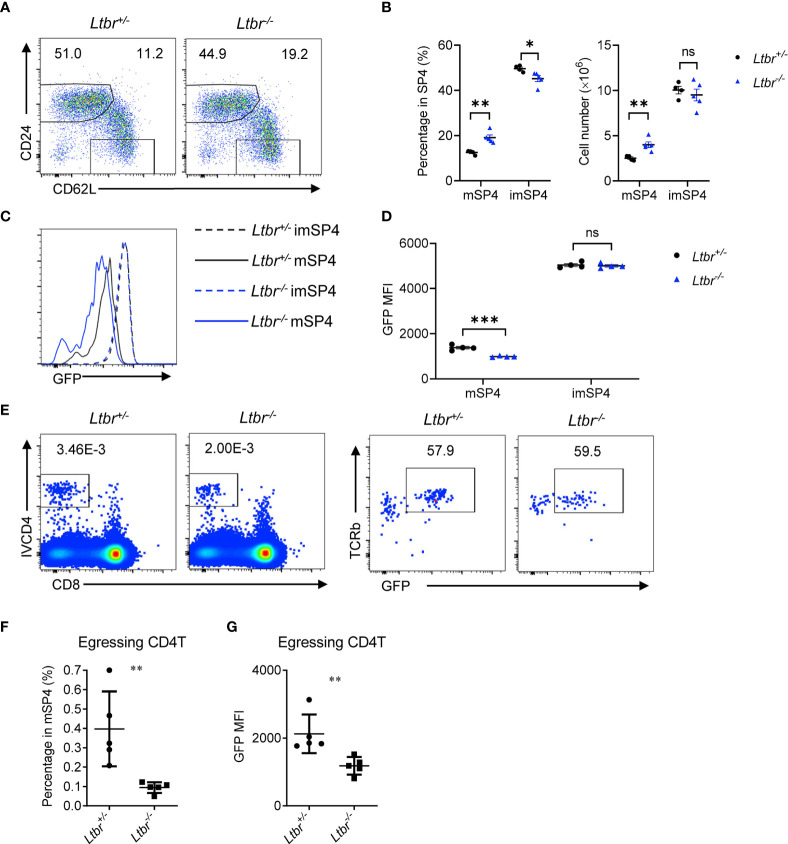
LTβR regulates thymic egress. **(A, B)** Flow cytometric analysis of single-positive (SP) CD4^+^ (SP4) thymocytes in *Ltbr* ^−/−^ and littermate control mice. **(A)** Representative dot plots are shown. **(B)** The graphs display the statistical analysis of the percentage of immature (imSP4) or mature (mSP4) thymocytes among total SP4 population. Mean ± SEM; n = 4 and 5. Data are representative of at least three independent experiments. **(C, D)** Flow cytometric analysis of GFP expression on SP4 thymocytes in *Ltbr* ^−/−^ Rag2pGFP and littermate control mice. **(C)** Representative histogram plots are shown. **(D)** The graphs display the statistical analysis of the GFP mean fluorescence intensity (MFI) of populations in **(C)** Mean ± SEM; n = 4. Data are representative of at least three independent experiments. **(E, F)** Flow cytometric analysis of IVCD4-labeled GFP^+^ thymocytes in *Ltbr* ^−/−^ and littermate control mice. **(E)** Representative dot plots are shown. **(F)** The graphs display the statistical analysis of the percentage of IVCD4-labeled GFP^+^ thymocytes among total mSP4. **(G)** Flow cytometric analysis of GFP expression on IVCD4-labeled GFP^+^ thymocytes in *Ltbr* ^−/−^ Rag2pGFP and littermate control mice. The graphs display the statistical analysis of GFP MFI of egressing CD4^+^ T cells. Mean ± SEM; n = 5. Data are representative of at least three independent experiments. ns, P > 0.05; *P < 0.05; **P < 0.01; ***P < 0.001 (unpaired Student’s *t*-test).

To examine the role of LTβR signaling in thymic egress in more details, we first detected thymocyte populations in *Ltbr* ^−/−^ mice. Consistent with the previous work (8, 9), mature thymocytes, defined as CD62L^+^CD24^−^ (**Figures 1A, B**) or CD62L^+^CD69^−^ (**Supplementary Figures 1A, B**), were remarkably accumulated in *Ltbr* ^−/−^ mice compared with littermate *Ltbr* ^+/−^ control mice, indicating a deficiency in thymic egress in *Ltbr* ^−/−^ mice. Meanwhile, immature thymocytes, defined as CD62L^−^CD24^hi^ or CD62L^−^CD69^+^, were comparable between *Ltbr* ^−/−^ and littermate control mice, suggesting this is not due to accelerated thymocyte maturation. To further verify this, we crossed *Ltbr* ^−/−^ mice with Rag2pGFP mice, where GFP intensity acted as a “molecular timer” to follow T cell development (10). We found that GFP intensity of mature thymocytes in *Ltbr* ^−/−^ Rag2pGFP mice is significantly lower than that in *Ltbr* ^+/−^ Rag2pGFP mice, while GFP intensity of immature thymocytes is comparable. These suggest a longer stay of mature thymocytes in the thymus of *Ltbr* ^−/−^ Rag2pGFP mice (**Figures 1C, D** and **Supplementary Figure 1D**). A previous study has ruled out abnormally higher proliferation of mature thymocytes in *Ltbr* ^−/−^ mice by *in vivo* BrdU labeling (9). To directly evaluate thymic egress of *Ltbr* ^−/−^ Rag2pGFP mice, we adopted the intravascular thymocyte labeling assay as previously described (5) to detect egressing thymocytes. One μg CD4-PE (IVCD4) antibody was intravenously injected per mouse. Three to 5 min later, the mice were sacrificed and thymocytes were prepared for staining and flow cytometric analysis. GFP intensity was also determined to discriminate between egressing thymocytes and recirculating T cells. Significantly fewer egressing thymocytes were found in *Ltbr* ^−/−^ Rag2pGFP mice (**Figures 1E, F**). Meanwhile, the GFP intensity of egressing thymocytes was lower in *Ltbr* ^−/−^ Rag2pGFP mice compared to those in *Ltbr* ^+/−^ Rag2pGFP mice (**Figure 1G**). Together, these data suggest that LTβR signaling indeed regulates thymic egress.

### EC-Derived LTβR Signaling Regulates Thymic Egress

**Figure 2:**
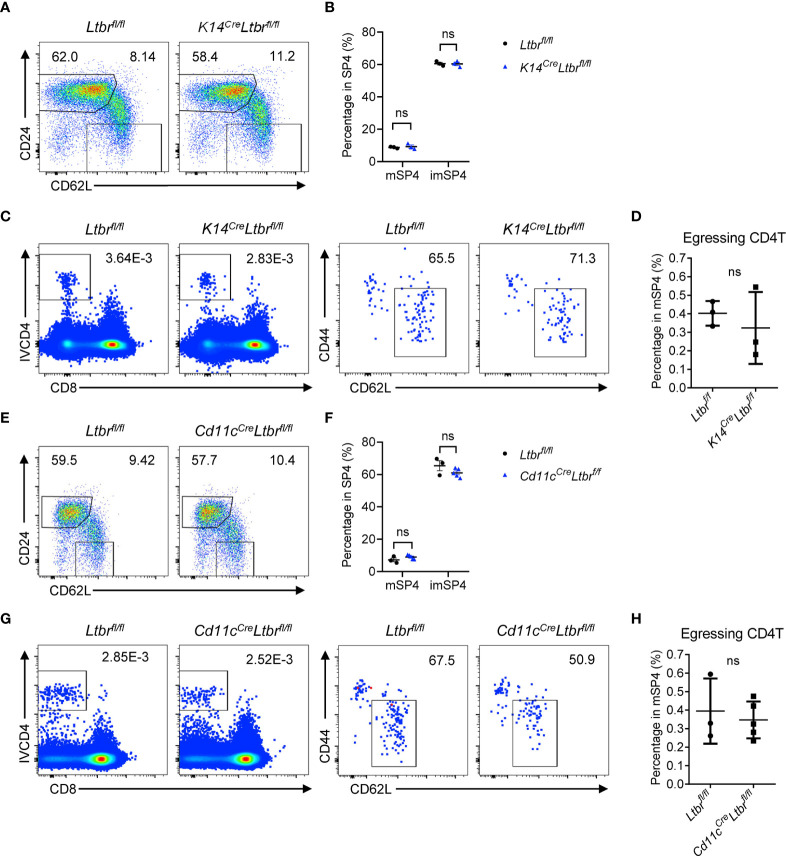
TEC- or DC-derived LTβR does not regulate thymic egress. **(A, B)** Flow cytometric analysis of SP4 thymocytes in *K14* ^Cre^ *Ltbr* ^fl/fl^ and littermate control mice. **(A)** Representative dot plots are shown. **(B)** The graphs display the statistical analysis of the percentage of immature or mature CD4^+^ SP thymocytes among total SP4 population. Mean ± SEM; n = 3. Data are representative of at least three independent experiments. **(C, D)** Flow cytometric analysis of egressing CD4^+^ T cells in *K14* ^Cre^ *Ltbr* ^fl/fl^ and littermate control mice. **(C)** Representative dot plots of IVCD4-labeled thymocytes are shown (left). Egressing CD4^+^ T cells are gated from IVCD4-labeled thymocytes (right). **(D)** The graphs display the statistical analysis of the percentage of egressing CD4^+^ T cells among total mSP4. Mean ± SEM; n = 3. Data are representative of at least three independent experiments. **(E, F)** Flow cytometric analysis of SP4 thymocytes in *Cd11c* ^Cre^ *Ltbr* ^fl/fl^ and littermate control mice. **(E)** Representative dot plots are shown. **(F)** The graphs display the statistical analysis of the percentage of immature or mature thymocytes among total SP4 population. Mean ± SEM; n = 3 and 5. Data are representative of two independent experiments. **(G, H)** Flow cytometric analysis of egressing CD4^+^ T cells in *Cd11c* ^Cre^ *Ltbr* ^fl/fl^ and littermate control mice. **(G)** Representative dot plots of IVCD4-labeled thymocytes are shown (left). Egressing CD4^+^ T cells are gated from IVCD4-labeled thymocytes (right). **(H)** The graphs display the statistical analysis of the percentage of egressing CD4^+^ T cells. Mean ± SEM; n = 3 and 5. Data are representative of at least three independent experiments. ns, P > 0.05 (unpaired Student’s *t*-test).

To clarify the mechanism of LTβR signaling regulating thymic egress, we first investigated which cell type expressing LTβR is involved in thymic egress. LTβR expression has been reported in many cell components in thymus (11). Boehm et al. supposed that impaired lympho-epithelial crosstalk in the absence of LTβR signaling might impede mature thymocytes (9). To directly determine the role of epithelial LTβR in thymic egress, we generated *K14* ^Cre^ *Ltbr* ^fl/fl^ mice (12). The development and function of thymic epithelial cells (TECs) were significantly impaired in these mice (12). However, comparable population of mature thymocytes was found between *K14* ^Cre^ *Ltbr* ^fl/fl^ mice and *Ltbr* ^fl/fl^ littermate control mice (**Figures 2A–D** and **Supplementary Figures 2A, B**). Thus, TEC-derived LTβR signaling is not involved in thymic egress.

Thymic DCs play an essential role in thymic egress through expressing sphingosine-1-phosphate (S1P) lyase to maintain the localized S1P gradient in the thymus (13). The homeostasis and function of DCs were affected by LTβR signaling (14). To test this hypothesis, *CD11c* ^Cre^ *Ltbr* ^fl/fl^ mice were generated. Again, no indication of thymic egress defect was found in these mice (**Figures 2E–H** and **Supplementary Figures 2C, D**).

**Figure 3:**
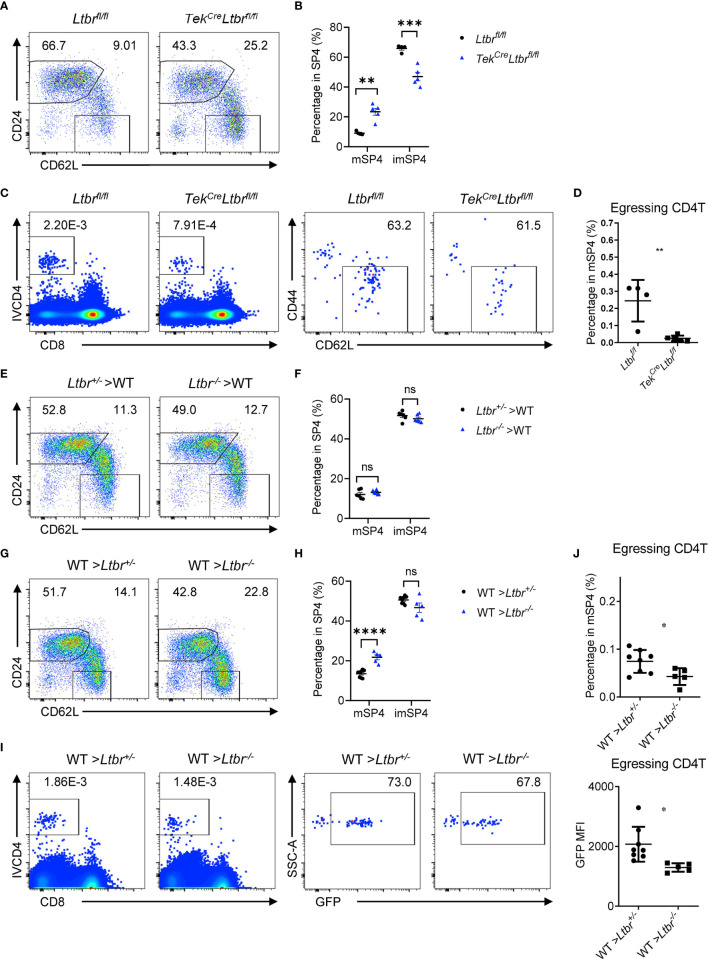
EC-derived LTβR regulates thymic egress. **(A, B)** Flow cytometric analysis of SP4 thymocytes in *Tek* ^Cre^ *Ltbr* ^fl/fl^ and littermate control mice. **(A)** Representative dot plots are shown. **(B)** The graphs display the statistical analysis of the percentage of immature or mature CD4^+^ SP thymocytes among total SP4 population. Mean ± SEM; n = 4 and 5. Data are representative of at least three independent experiments. **(C, D)** Flow cytometric analysis of egressing CD4^+^ T cells in *Tek* ^Cre^ *Ltbr* ^fl/fl^ and littermate control mice. **(C)** Representative dot plots of IVCD4-labeled thymocytes are shown (left). Egressing CD4^+^ T cells are gated from IVCD4-labeled thymocytes (right). **(D)** The graphs display the statistical analysis of the percentage of egressing CD4^+^ T cells among total mSP4. Mean ± SEM; n = 4 and 5. Data are representative of at least three independent experiments. **(E, F)** Flow cytometric analysis of SP4 thymocytes in bone marrow chimeric mice with LTβR deficiency in hematopoietic cells. **(E)** Representative dot plots are shown. **(F)** The graphs display the statistical analysis of the percentage of immature or mature CD4^+^ thymocytes among total SP4 population. Mean ± SEM; n = 6. Data are representative of at least three independent experiments. **(G**–**J)** Flow cytometric analysis of thymocytes in bone marrow chimeric mice with LTβR deficiency in irradiation-resistant cells. **(G)** Representative dot plots of SP4 thymocytes are shown. **(H)** The graphs display the statistical analysis of the percentage of immature or mature thymocytes. **(I)** Representative dot plots of IVCD4-labeled thymocytes are shown (left). Egressing CD4^+^ T cells are gated from IVCD4-labeled thymocytes (right). **(J)** The graphs display the statistical analysis of the percentage (top) and GFP MFI (bottom) of egressing CD4^+^ T cells. Mean ± SEM; n = 8 and 5. Data are representative of at least three independent experiments. ns, P > 0.05; *P < 0.05; **P < 0.01; ***P < 0.001; ****P < 0.0001 (unpaired Student’s *t*-test).

LTβR signaling regulates differentiation and function of HECs (7, 15, 16). Our previous work showed that LTβR is required for the development of TPECs involving in HPC homing (6). Tek^Cre^ *Ltbr* ^fl/fl^ mice were examined, and significant accumulation of mature thymocytes and reduced egressing thymocytes were found in these mice (**Figures 3A–D** and **Supplementary Figures 3A, B**), recapitulating the phenotype of *Ltbr* ^−/−^ mice, consistent with the recent study (8). Since *Tek* ^Cre^ also deletes floxed genes from hematopoietic cells, *Ltbr* ^−/−^ bone marrow chimeric mice were also generated. Normal population of mature thymocytes were determined when LTβR was deficient from hematopoietic cells (**Figures 3E, F**), while accumulated population of mature thymocytes and reduced population of egressing thymocytes were found when LTβR was deficient from irradiation-resistant cells (**Figures 3G–J**). Together, these data suggest that EC-derived LTβR is required for thymic egress.

### LT and LIGHT Expressed on T Cells Redundantly Regulate Thymic Egress

**Figure 4:**
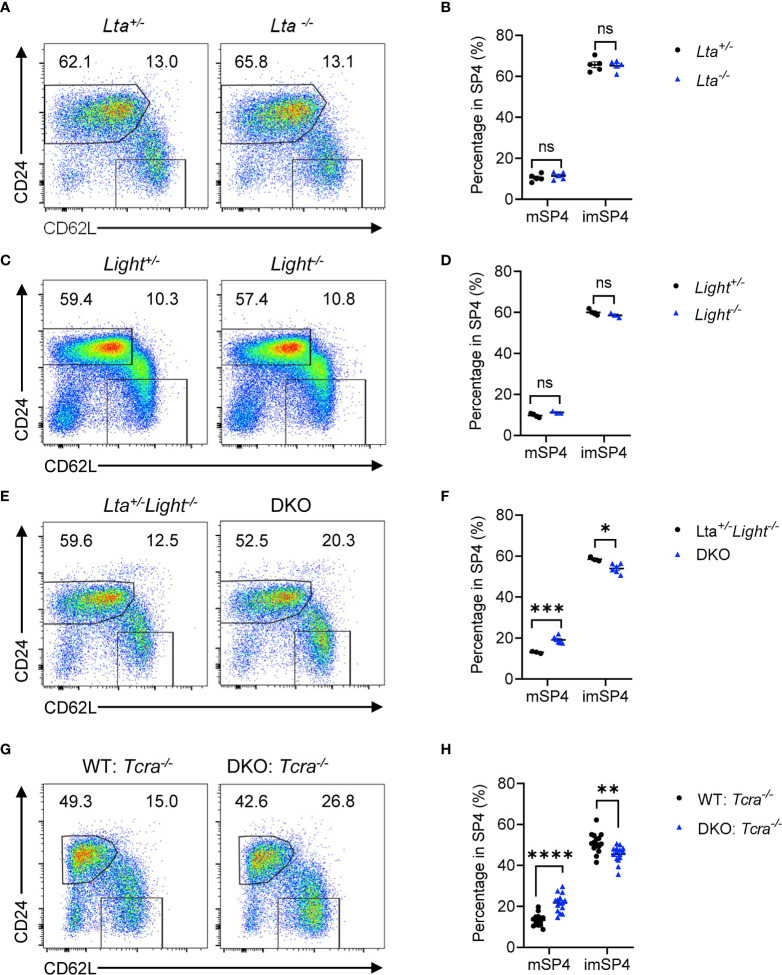
LT and LIGHT expressed on T cells redundantly regulate thymic egress. **(A, B)** Flow cytometric analysis of SP4 thymocytes in *Lta* ^−/−^ and littermate control mice. **(A)** Representative dot plots are shown. **(B)** The graphs display the statistical analysis of the percentage of immature or mature CD4^+^ SP thymocytes among total SP4 population. Mean ± SEM; n = 5. Data are representative of at least three independent experiments. **(C, D)** Flow cytometric analysis of SP4 thymocytes in *Light* ^−/−^ and littermate control mice. **(C)** Representative dot plots are shown. **(D)** The graphs display the statistical analysis of the percentage of immature or mature CD4^+^ SP thymocytes among total SP4 population. Mean ± SEM; n = 4 and 3. Data are representative of at least three independent experiments. **(E, F)** Flow cytometric analysis of SP4 thymocytes in *Lta* ^−/−^ *Light* ^−/−^ and littermate control mice. **(E)** Representative dot plots are shown. **(F)** The graphs display the statistical analysis of the percentage of immature or mature CD4^+^ SP thymocytes among total SP4 population. Mean ± SEM; n = 3 and 6. Data are representative of at least three independent experiments. **(G, H)** Flow cytometric analysis of thymocytes in bone marrow chimeric mice with deficiency of both LTβR ligands in T cells. **(G)** Representative dot plots of SP4 thymocytes are shown. **(H)** The graphs display the statistical analysis of the percentage of immature or mature CD4^+^ SP thymocytes among total SP4 population. Mean ± SEM; n = 15. Data are pooled from three independent experiments. ns, P > 0.05; *P < 0.05; **P < 0.01; ***P < 0.001; ****P < 0.0001 (unpaired Student’s *t*-test).

LTβR has two ligands, LT and LIGHT, expressed on various cell types respectively, involved in multiple physiological processes. To evaluate which ligand is required for thymic egress, we detected thymocyte population of *Lta* ^−/−^ and *Light* ^−/−^ mice respectively. We found comparable mature thymocyte population in solely *Lta* ^−/−^ or *Light* ^−/−^ mice compared to their heterozygous littermate controls (**Figures 4A–D** and **Supplementary Figures 4A–D**). We asked whether LT and LIGHT might play redundant roles regulating thymic egress. To test this hypothesis, we crossbred and obtained *Lta* ^−/−^ *Light* ^−/−^ mice (double knockout, DKO). We found remarkable accumulation of mature thymocytes and reduced population of egressing thymocytes in DKO mice compared to the littermate *Lta* ^+/−^ *Light* ^−/−^ mice (**Figures 4E, F** and **Supplementary Figures 4E, F**). Thus, LT and LIGHT redundantly regulate thymic egress.

To further clarify the cellular interaction of LTβR signaling in thymic egress, we wondered which cell type delivers ligand signals. Our previous work demonstrated that T cell-EC mediated LT/LIGHT-LTβR signaling regulated HPC homing (6). Here, to determine whether T cells deliver LTβR ligands to regulate thymic egress, we constructed mixed bone marrow chimeric mice with WT : T*cra* ^−/−^ or DKO : *Tcra* ^−/−^ mixed bone marrow cells at a ratio of 2:8 in irradiated WT hosts as described previously (6). Six weeks later, chimeric mice were examined. Remarkable accumulation of mature thymocytes and reduction of egressing thymocytes were found in mice with T cell conditional ligands deficiency (**Figures 4G, H** and **Supplementary Figures 4G–J**), suggesting that T cells deliver LTβR ligands to guide thymic egress.

### TPECs Serve as a Gatekeeper for Thymic Egress

**Figure 5:**
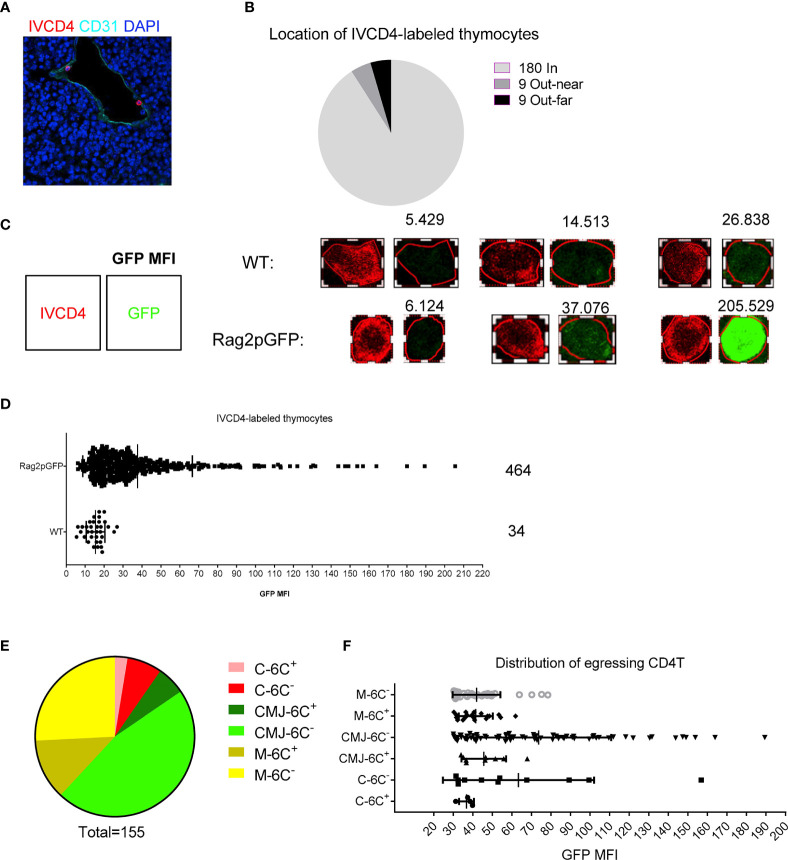
Mature thymocytes emigrate through TPECs. **(A)** Representative image of IVCD4-labeled thymocytes is shown. **(B)** Graph displays the statistical analysis of the location of IVCD4-labeled thymocytes as in **(A)** Data from more than five experiments were pooled for statistical analysis, n = 198. (**C**–**F)** Location of egressing CD4 T^+^ cells. **(C)** Defined the cell outline according to the CD4-PE signal (left), and numbers (upper) showed the calculated GFP MFI of the cell (right). Three representative cells with different GFP level are shown for each group. **(D)** Statistical analysis of the GFP MFI of IVCD4-labeled thymocytes in Rag2pGFP or WT mice. **(E)** Statistical analysis of the location of 155 egressing CD4^+^ T cells. C, cortex; M, medulla; CMJ, cortico-medullary junction; 6C^+^, Ly6C^+^ vessels; 6C^−^, Ly6C^−^ vessels. **(F)** GFP MFI of the egressing CD4^+^ T cells, grouped by the location.

These previous results have uncovered a cellular and molecular mechanism for thymic egress control similar to that for TPEC control of thymic homing as we described previously (6). We wondered about the relationship between TPECs and thymic egress. To address this question, we again employed the intravascular labeling assay to locate egressing thymocytes in the thymus (5). CD31 was used to define thymic blood vessels. Most of IVCD4-labeled thymocytes were found to be located in vessels (180/198) and few located out of vessels, near or far from vessels (**Figures 5A, B**), suggesting successful labeling of thymic CD4^+^ T cells exposed to the vascular compartment. To distinguish egressing thymocytes from recirculating T cells in vessels, we did the same experiment in Rag2pGFP mice. We used CD4-PE signal to define the outline of IVCD4-labeled thymocytes and analyzed the GFP intensity of the cell (**Figure 5C**). Compared with IVCD4-labeled thymocytes in WT mice (most cells with GFP MFI under 20), IVCD4-labeled thymocytes in Rag2pGFP mice showed broad distribution of GFP intensity (**Figure 5D**). Referring to the GFP intensity in WT mice, we set the threshold, MFI = 30, to distinguish GFP^+^ egressing thymocytes from GFP^-^ recirculating T cells. Meanwhile, we used 50 μm as the distance to identify the range of CMJ. Statistical analysis showed that egressing thymocytes are mainly located at Ly6C^−^ vessels at CMJ or medulla where HPCs enter (**Figures 5E, F**). Thus, in addition to serving as the entry site for HPCs (6), Ly6C^−^ ECs (TPECs) appear to also serve as the exit site for mature thymocytes.

### TPECs Are Heterogeneous

**Figure 6:**
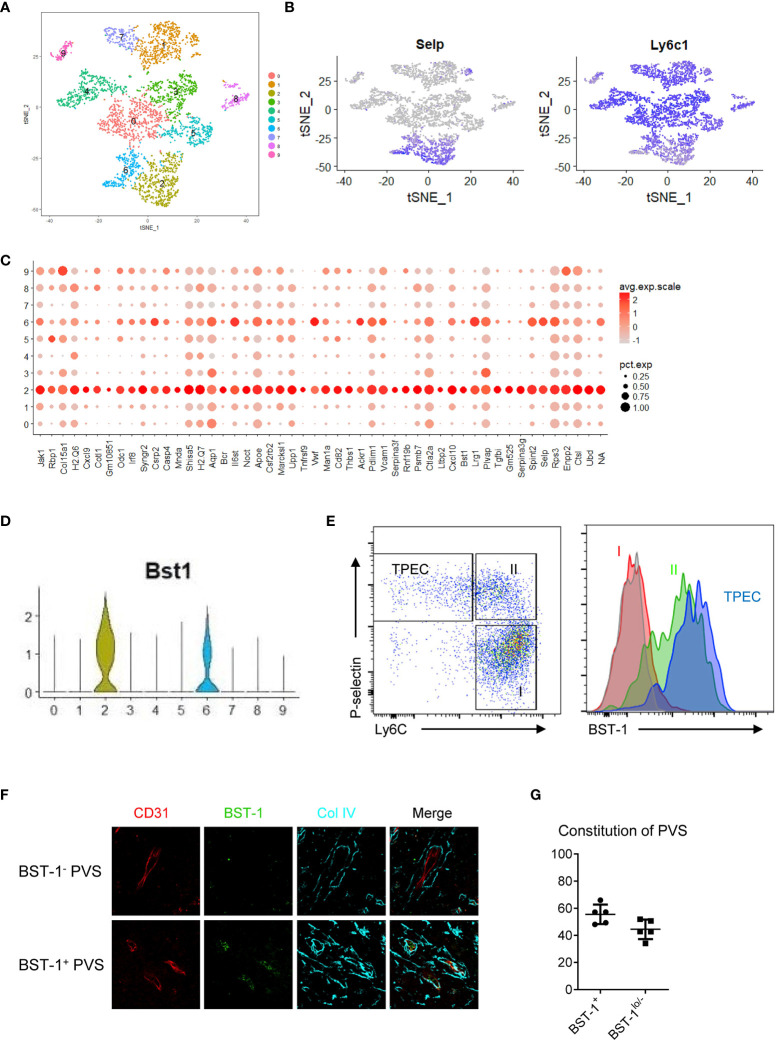
TPECs are heterogeneous and can be divided into two subsets *via* BST-1 expression level. **(A)** t-SNE analysis of scRNA-seq data from total thymic ECs readily divide thymic ECs into 10 clusters. **(B)** Transcriptional level of P-selectin (left) and Ly6C (right) in the thymic ECs. **(C)** Signature genes of cluster 2 (C2). **(D)** Violin plot analysis of BST-1 gene expression on different clusters of thymic ECs. **(E)** Flow cytometric analysis of BST-1 protein expression on major subsets of thymic ECs. Left: gating strategy. Subset I: Ly6C^+^P-selectin^−^; Subset II: Ly6C^+^P-selectin^+^; TPEC: Ly6C^-^P-selectin^−^. Right: histogram of BST-1 expression. Gray: isotype control; Red: Subset I; Green: Subset II; Purple: TPEC. **(F)** BST-1 expression on vessels within PVS. Representative photos of PVS surrounding vessels with different expression levels of BST-1 are shown. **(G)** Statistical analysis of BST-1^+^ and BST-1^lo/−^ PVS surrounding vessels.

TPECs are the HEC-like population in the thymus (6). While HECs are the entry sites for naïve B and T cells in LNs, their exit sites are at the cortical sinus (17). The entry and exit sites for lymphocytes in LNs are distinct. Herein, we found that TPECs undertook dual roles as both entry and exit sites. We wondered whether TPECs might be heterogeneous and different subsets of TPECs might exist, controlling different trafficking behaviors. To do this, scRNA-seq was applied to thymic ECs (**Supplementary Figure 5**). A total of 5,003 thymic ECs from WT mice were analyzed, and the mean read per cell is 87,654, and the median number of genes per cell is 2,276. Interestingly, tSNE analysis showed that thymic ECs are actually highly heterogenous (**Figure 6A**). Among them, TPECs were clearly defined, confirming their unique presence (**Figure 6B**). More importantly, TPECs can be further divided into two clusters, C2 and C6. To further define these TPEC subsets, we analyzed the signature genes of C2 and C6.

Claudin 5 is a membrane protein of tight junctions, playing an important role in maintaining blood–brain barrier and blood–thymus barrier (18, 19). In the thymus, Claudin 5-negative thymic ECs were found associated with thymic entry of bloodborne molecules, such as S1P, and thymic egress of mature thymocytes (19). Interestingly, we found that Claudin 5 is downregulated in both C2 and C6 subsets (**Supplementary Figure 6A**), consistent with their function as TPECs.

Chemokines play important roles for thymic homing of HPCs and egress of mature thymocytes (20, 21). We found both Cxcl9 and Cxcl10 were selectively expressed in C2 rather than in C6 or other subsets (**Supplementary Figures 6B, C**). In fact, Cxcr3, the receptor of Cxcl9/10, is just highly expressed on the most mature thymocytes (21, 22), suggesting a possibility of thymic egress regulation *via* Cxcl9/10-Cxcr3 signaling axis. These suggest that C2 subset might be a source of chemokines to guide mature thymocytes for their egress.

S1P is a critical factor controlling thymic egress of mature thymocytes (23). Sphk1, coding sphingosine kinase, was found expressed at a higher level in C2 than in C6 subset (**Supplementary Figure 6D**). Sphk1 has been reported as an important factor controlling S1P production (23). These might suggest C2 subset might be involved in thymic egress *via* local production of S1P.

To further test whether C2 subset might be indeed associated with thymic egress, we looked for C2 specific surface markers for better identification. Among the C2 signature genes (**Figure 6C**), BST-1, a surface molecule, is preferentially expressed on C2 rather than C6, but not on other thymic ECs (**Figure 6D**). Flow cytometry confirmed its specific expression on thymic ECs, particularly on TPECs (**Figure 6E**). While no distinct subsets relative to C2 and C6 were seen according to BST-1 expression by flow cytometry, immunofluorescence microscopy showed BST-1^+^ or BST-1^−^ associated PVSs (**Figure 6F**), probably due to less sensitivity of microscopy than flow cytometry. Further immunofluorescence staining analysis of thymic sections from WT mice revealed about 60% PVSs surrounding BST-1^hi^ vessels while 40% PVSs surrounding BST-1^lo/−^ vessels (**Figure 6G**). Together, these suggested that TPECs might be divided into two functional subsets based on its expression level: BST-1^hi^ or BST-1^lo/−^.

### BST-1^hi^ TPECs Function as the Exit Site for Mature Thymocytes, While BST-1^lo/−^ TPECs Serve as the Entry Sites for HPCs

**Figure 7:**
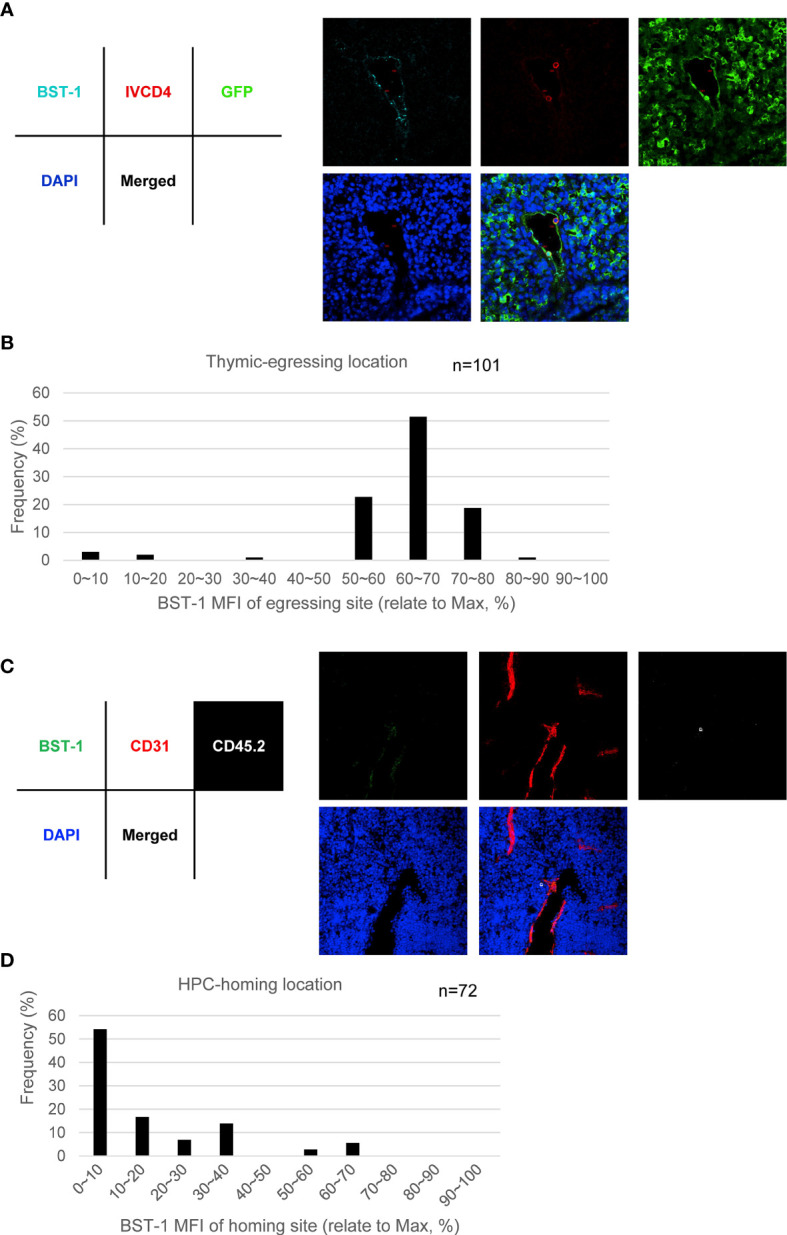
BST-1^hi^ TPECs function as the exit sites for mature thymocytes, while BST-1^lo/−^ TPECs serve as the entry sites for HPCs. **(A, B)** Egressing CD4^+^ T cells are mainly associated with BST-1^hi^ vessels. **(A)** Representative photo of egressing CD4^+^ T cells locate at BST-1^hi^ vessel. **(B)** Location distribution of egressing CD4^+^ T cells in relative to BST-1 MFI of the vessels. Totally 101 egressing CD4^+^ T cells were collected and calculated. **(C, D)** Thymic-settling HPCs are mainly associated with BST-1^lo/−^ vessels. **(C)** Representative photo of homing HPCs locate at BST-1^lo/−^ vessels. **(D)** Location distribution of settling HPCs in relation to BST-1 MFI of the vessels. Totally 72 settling HPCs were collected and calculated.

To further examine the function of BST-1^hi^ or BST-1^lo/−^ TPECs on thymic trafficking, we reanalyzed the position of egressing thymocytes relative to BST-1 expression. Interestingly, most egressing thymocytes were found located at BST-1^hi^ vessels, suggesting a labor division of these two subsets of TPECs (**Figures 7A, B**). To further determine whether BST-1^lo/−^ might be related to HPC homing, a short-term thymic homing assay was performed with sorted lineage^-^c-Kit^+^ bone marrow cells. Indeed, the majority of thymic seeding progenitor cells were located close to the BST-1^lo/−^ vessels (**Figures 7C, D**). Thus, these results demonstrated that mature thymocytes exit and HPCs entry take place at different vessels: BST-1^hi^ vessels for emigration while BST-1^lo/−^ vessels for homing. They are therefore named emigration-associated TPEC (eTPECs) and immigration-associated TPEC (iTPECs), respectively.

### BST-1 Does Not Regulate Thymic Egress

**Figure 8:**
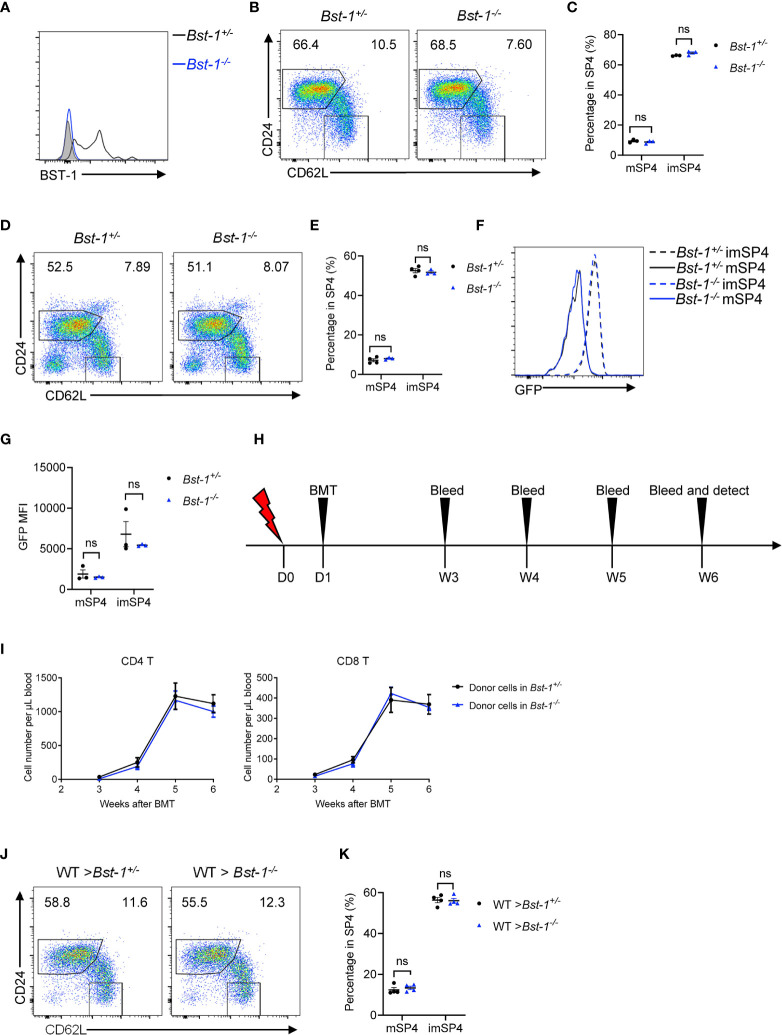
BST-1 does not regulate thymic egress. **(A)** Flow cytometric analysis of BST-1 expression on thymic TPECs (CD45^−^CD31^+^Ly6C^−^P-selectin^+^) in *Bst-1* ^−/−^ and littermate control mice. **(B, C)** Flow cytometric analysis of SP4 thymocytes in young (4–6 weeks old) *Bst-1* ^−/−^ and littermate control mice. **(B)** Representative dot plots are shown. **(C)** The graphs display the statistical analysis of the percentage of immature or mature CD4^+^ SP thymocytes among total SP4 population. Mean ± SEM; n = 3. Data are representative of at least three independent experiments. **(D, E)** Flow cytometric analysis of SP4 thymocytes in aged (30 weeks old) *Bst-1* ^−/−^ and littermate control mice. **(D)** Representative dot plots are shown. **(E)** The graphs display the statistical analysis of the percentage of immature or mature CD4^+^ SP thymocytes among total SP4 population. Mean ± SEM; n = 4 and 3. Data are representative of two independent experiments. **(F, G)** Flow cytometric analysis of GFP expression on SP4 thymocytes in *Bst-1* ^−/−^ Rag2pGFP and littermate control mice. **(F)** Representative histogram plots are shown. **(G)** The graphs display the statistical analysis of the GFP MFI of populations in **(F)** Mean ± SEM; n = 3. Data are representative of at least three independent experiments. **(H**–**K)** Flow cytometric analysis of thymic egress in mice with Bst-1 deficiency in radioresistant cells. **(H)** Work model of bone marrow reconstitution and detection. **(I)** The graphs display the statistical analysis of the numbers of donor-derived CD4^+^ or CD8^+^ T cells in the blood of bone marrow chimeric mice. Mean ± SEM; n = 4. Data are representative of two independent experiments. **(J)** Flow cytometric analysis of SP4 thymocytes in bone marrow chimeric mice. Representative dot plots are shown. **(K)** The graphs display the statistical analysis of the percentage of immature or mature CD4^+^ SP thymocytes among total SP4 population. Mean ± SEM; n = 4. Data are representative of two independent experiments. ns, P > 0.05 (unpaired Student’s *t*-test).

Since BST-1 provided us with an appropriate marker for discriminating entry and exit site of thymus, it is worth to investigate whether BST-1 regulates thymic egress. BST-1, like CD38, behaves both as an ectoenzyme and signaling receptor and has been reported to regulate the trafficking of neutrophil and monocytes (24). We generated and detected *Bst-1* ^−/−^ mice and found no accumulation of mature thymocytes in young *Bst-1* ^−/−^ mice (**Figures 8A–C**). This does not change in aged *Bst-1* ^−/−^ mice (**Figures 8D, E**). Fine determination of GFP intensity of mature thymocytes further confirmed the normal thymic egress in *Bst-1* ^−/−^ Rag2pGFP (**Figures 8F, G**). To exclude the potential influence of BST-1 in hematopoietic cells, we have also constructed bone marrow chimeric mice. Still, no indication of thymic egress defect was found in mice with Bst-1 deficiency in radioresistant cells (**Figures 8H–K**). These results together suggested that although BST-1 might be used as a marker to characterize eTPECs, it probably does not regulate its function.

### LTβR Signaling and T Cells Regulate the Development of eTPECs

**Figure 9:**
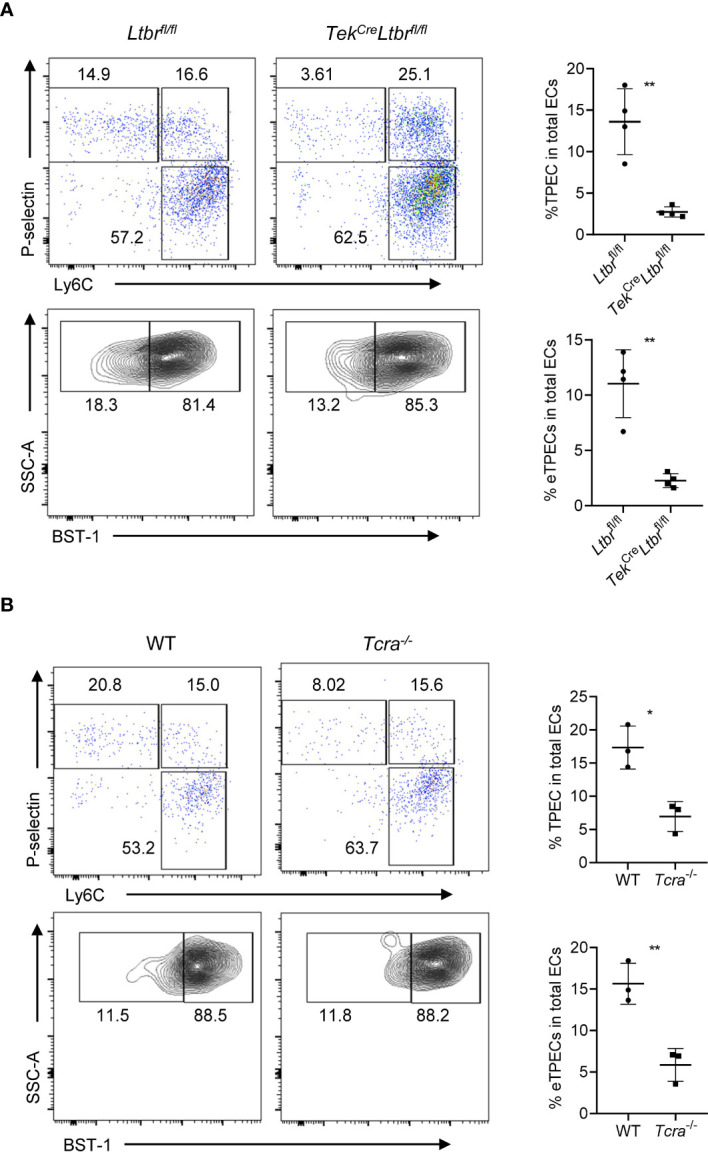
LTβR signaling and T cells regulate the development of eTPECs. **(A)** Flow cytometric analysis of TPEC and eTPECs in *Ltbr* ^fl/fl^ and *Tek* ^Cre^ *Ltbr* ^fl/fl^ mice. Left: Representative plots are shown. Right: Graphs display the statistical analysis of the percentage of TPECs or eTPECs. Mean ± SEM; n = 4. Data are representative of at least three independent experiments. **(B)** Flow cytometric analysis of TPEC and eTPECs in WT and *Tcra* ^−/−^ mice. Left: Representative plots are shown. Right: Graphs display the statistical analysis of the percentage of TPECs or eTPECs. Mean ± SEM; n = 3. Data are representative of two independent experiments. *P < 0.05; **P < 0.01 (unpaired Student’s *t*-test).

Our previous study has found the critical role of LTβR signaling on TPEC development as a whole population (6). To study whether eTPECs are also regulated by LTβR, we more specifically detected the BST-1^hi^ subset in total TPEC population in LTβR conditional knockout mice. Indeed, consistent with the reduction of total TPEC population, the eTPEC subset is also significantly reduced in *Tek* ^Cre^ *Ltbr* ^fl/fl^ mice (**Figure 9A**). We have also previously found that T cells regulate thymic TPECs (6). To test further whether this might be also true for eTPECs, *Tcra^−/−^* mice were examined, and the eTPEC subset was also found significantly reduced in *Tcra^−/−^* mice compared to that in WT mice (**Figure 9B**). Thus, these data suggest that T cell–derived signal might engage endothelial LTβR for the development/homeostasis of eTPECs. However, whether it is the LT/LIGHT signal derived from T cells that controls eTPECs still remains to be determined using conditionally gene-deficient mice in future.

## Discussion

Mature thymocytes immigrate to periphery through thymic blood vessel (5). However, the precise definition and characterization of the cellular basis of this process are poorly understood. In this study, using conventional and conditional gene-deficient mouse models, we revealed the cellular mechanism of LT/LIGHT-LTβR-mediated T-EC crosstalk in regulating thymic egress. Further, scRNA-seq revealed different subsets of TPECs controlling thymic homing of HPCs and egress of mature thymocytes, respectively.

Thymocyte-thymic stroma crosstalk plays important roles during thymocyte development (25–27). Although the crosstalk mechanism for thymocyte development and selection has been extensively studied (25–27), how the crosstalk is involved in thymic homing and egress is less clear. Our previous study has revealed a crosstalk mechanism between T cells and ECs controlling thymic homing of HPCs (6). Here, our data further suggested the crosstalk between T cells and ECs is also important for thymic egress.

LT/LIGHT-LTβR is a key signaling axis mediating diverse crosstalk in the thymus. LTβR is widely expressed on stromal cells including epithelial cells, mesenchymal stromal cells, ECs, etc., and non-lymphoid immune cells, such as DCs. An early study proposed that the impaired thymic egress in LTβR-deficient mice might be due to impaired LTβR signaling in thymic epithelial cells (9), but no direct evidence was provided. We have previously found that epithelial LTβR signaling is indeed required for medullary thymic epithelial cell development (12). Reduced expression of chemokines, such as Ccl19/Ccl21, in medullary thymic epithelial cells was also found in LTβR-deficient mice (28). However, our current data suggest that the impaired medullary thymic epithelial development and function in the absence of LTβR signaling do not seem to influence mature thymocyte thymic egress. This was also supported by a previous study (8). In addition to the regulation of medullary thymic epithelial cells, LTβR signaling has also been well documented to control thymic mesenchymal stromal cells (29), a key regulator of thymic egress (5). However, clear evidence has also ruled out the possibility of mesenchymal LTβR for thymic egress regulation (8). Another cell population influenced by LTβR and also involved in thymic egress is DCs (13, 30, 31). However, conditional LTβR deficiency in DCs does not impair thymic egress, either, as has been shown in our current study. Thus, together with the previous study (8), our current work has excluded the role of LTβR on thymic epithelial cells, mesenchymal stromal cells, and DCs, and has further confirmed the essential role of LTβR signaling derived from ECs for the regulation of thymic egress (12, 28).

As to the signal source of LTβR signaling, our study suggests LT/LIGHT derived from positively selected T cells. Using conditional knockout mouse model, we have found that double deficiency of LT and LIGHT on positively selected T cells results in significant impairment of thymic egress, comparable to those found in global LTβR-deficient mice or endothelial LTβR-deficient mice. LTα and LTβ expression on thymocytes increases after positive selection, especially in SP4 thymocytes, while thymocytes retain LIGHT expression over development in the thymus (32). However, whether the ligand signal is derived from mature thymocytes *per se* or recirculating T cells from periphery remains to be determined. It has been reported that effector/memory T cells from periphery are able to reenter the thymus through the CMJ area (33), and they have comparable expression of LTα and LTβ and even higher expression of LIGHT than those on mature thymocyte (32). It should be noted that the number of recirculating T cells are gradually enhanced during mice aging (34). In our study, 6–8-week-old mice were routinely utilized, and obvious defects of thymic egress have been detected. These might indicate a prominent role of thymocyte-derived LT/LIGHT signal to control their egress. Whether recirculating T cells may play more important roles in aged mice is an intriguing question for further investigation.

Previous data suggested that positively selected thymocytes control TPECs *via* the same LT/LIGHT-coordinated signals during thymic progenitor cell homing (6), indicating that both HPC homing and mature thymocyte egress may occur at TPECs. Immunofluorescence staining analysis of egressing thymocytes confirmed that TPECs also serve as the exit sites for mature thymocytes. Thus, thymus entry and exit, both regulated by LTβR signaling, could be linked by TPECs. scRNA-seq analysis of thymic ECs confirmed the unique presence of TPECs as we previously found (6). Interestingly, TPECs can be further divided into two subsets (C2 and C6) according to BST-1 expression. We reanalyzed the relevance between BST-1^hi^ or BST-1^lo/−^ TPECs and thymus entry or exit, respectively. Intriguingly, we found that BST-1^hi^ and BST-1^lo/−^ TPECs served as the exit sites and entry sites of thymus, respectively. We therefore redefined BST-1^hi^ TPECs as eTPECs, while BST-1^lo/−^ TPECs as iTPECs. The separation of these new subsets may shed new light to the mechanistic study of thymic trafficking. Indeed, further characterization has revealed some interesting molecules that are selectively expressed on eTPECs. These include chemokines Cxcl9 and Cxcl10, and sphingosine kinase Sphk1, which is important for S1P production. Given the selectively high expression of Cxcl9/10 receptor on the most mature thymocytes (21, 22) and the well-established role of S1P in thymic egress (23), these results might indicate a special local niche attracting mature thymocytes for their egress. In addition, a bigger question that remains largely elusive is how seemingly the same crosstalk between T cells and thymic ECs *via* the same LT/LIGHT-LTβR axis could control both thymic homing and egress *via* iTPECs and eTPECs, respectively. So far, there is still little clue present. More delicate studies are required to answer these interesting questions in the future.

It has been intriguing how thymic homing and egress both take place at PVS. Our current identification of iTPECs and eTPECs provides novel insight for further understanding these different trafficking processes.

## Data Availability Statement

The datasets presented in this study can be found in online repositories. The names of the repository/repositories and accession number(s) can be found in the article/**Supplementary Material**.

## Ethics Statement

The animal study was reviewed and approved by institutional committee of the Institute of Biophysics, Chinese Academy of Sciences.

## Author Contributions

HX designed and performed most experiments, analyzed the data, and wrote the manuscript. SZ performed scRNA-seq experiment and analyzed the data. YZ, BR, ZW, YS, and QC helped in some experiments. XW supervised the scRNA-seq experiment and analyzed the data. MZ conceived and supervised the project, analyzed the data, and wrote the manuscript. All authors contributed to the article and approved the submitted version.

## Conflict of Interest

The authors declare that the research was conducted in the absence of any commercial or financial relationships that could be construed as a potential conflict of interest.

## References

[1] Petrie HT Zúñiga-Pflücker JC. Zoned Out: Functional Mapping of Stromal Signaling Microenvironments in the Thymus. Annu Rev Immunol (2007) 25:649–79. 10.1146/annurev.immunol.23.021704.115715 17291187

[2] James KD Jenkinson WE Anderson G. T-Cell Egress From the Thymus: Should I Stay or Should I Go? J Leukoc Biol (2018) 104:275–84. 10.1002/JLB.1MR1217-496R PMC6174998 29485734

[3] Scimone ML Aifantis I Apostolou I von Boehmer H von Andrian UH. A Multistep Adhesion Cascade for Lymphoid Progenitor Cell Homing to the Thymus. Proc Natl Acad Sci U.S.A. (2006) 103:7006–11. 10.1073/pnas.0602024103 PMC1459009 16641096

[4] Mori K Itoi M Tsukamoto N Kubo H Amagai T. The Perivascular Space as a Path of Hematopoietic Progenitor Cells and Mature T Cells Between the Blood Circulation and the Thymic Parenchyma. Int Immunol (2007) 19:745–53. 10.1093/intimm/dxm041 17493961

[5] Zachariah MA Cyster JG. Neural Crest-Derived Pericytes Promote Egress of Mature Thymocytes at the Corticomedullary Junction. Science (2010) 328:1129–35. 10.1126/science.1188222 PMC3107339 20413455

[6] Shi Y Wu W Chai Q Li Q Hou Y Xia H. Ltβr Controls Thymic Portal Endothelial Cells for Haematopoietic Progenitor Cell Homing and T-cell Regeneration. Nat Commun (2016) 7:12369. 10.1038/ncomms12369 27493002 PMC4980457

[7] Moussion C Girard JP. Dendritic Cells Control Lymphocyte Entry to Lymph Nodes Through High Endothelial Venules. Nature (2011) 479:542–6. 10.1038/nature10540 22080953

[8] James KD Cosway EJ Lucas B White AJ Parnell SM Carvalho-Gaspar M. Endothelial Cells Act as Gatekeepers for Ltβr-Dependent Thymocyte Emigration. J Exp Med (2018) 215:2984–93. 10.1084/jem.20181345 PMC6279407 30425120

[9] Boehm T Scheu S Pfeffer K Bleul CC. Thymic Medullary Epithelial Cell Differentiation, Thymocyte Emigration, and the Control of Autoimmunity Require Lympho-Epithelial Cross Talk *Via* Ltbetar. J Exp Med (2003) 198:757–69. 10.1084/jem.20030794 PMC2194183 12953095

[10] McCaughtry TM Wilken MS Hogquist KA. Thymic Emigration Revisited. J Exp Med (2007) 204:2513–20. 10.1084/jem.20070601 PMC2118501 17908937

[11] Lu TT Browning JL. Role of the Lymphotoxin/LIGHT System in the Development and Maintenance of Reticular Networks and Vasculature in Lymphoid Tissues. Front Immunol (2014) 5:47. 10.3389/fimmu.2014.00047 24575096 PMC3920476

[12] Wu W Shi Y Xia H Chai Q Jin C Ren B. Epithelial Ltβr Signaling Controls the Population Size of the Progenitors of Medullary Thymic Epithelial Cells in Neonatal Mice. Sci Rep (2017) 7:44481. 10.1038/srep44481 28290551 PMC5349570

[13] Zamora-Pineda J Kumar A Suh JH Zhang M Saba JD. Dendritic Cell sphingosine-1-phosphate Lyase Regulates Thymic Egress. J Exp Med (2016) 213:2773–91. 10.1084/jem.20160287 PMC5110016 27810923

[14] Satpathy AT Briseño CG Lee JS Ng D Manieri NA Kc W. Notch2-dependent Classical Dendritic Cells Orchestrate Intestinal Immunity to Attaching-and-Effacing Bacterial Pathogens. Nat Immunol (2013) 14:937–48. 10.1038/ni.2679 PMC3788683 23913046

[15] Browning JL Allaire N Ngam-Ek A Notidis E Hunt J Perrin S. Lymphotoxin-Beta Receptor Signaling is Required for the Homeostatic Control of HEV Differentiation and Function. Immunity (2005) 23:539–50. 10.1016/j.immuni.2005.10.002 16286021

[16] Onder L Danuser R Scandella E Firner S Chai Q Hehlgans T. Endothelial Cell-Specific Lymphotoxin-β Receptor Signaling is Critical for Lymph Node and High Endothelial Venule Formation. J Exp Med (2013) 210:465–73. 10.1084/jem.20121462 PMC3600902 23420877

[17] Girard JP Moussion C Förster R. Hevs, Lymphatics and Homeostatic Immune Cell Trafficking in Lymph Nodes. Nat Rev Immunol (2012) 12:762–73. 10.1038/nri3298 23018291

[18] Greene C Hanley N Campbell M. Claudin-5: Gatekeeper of Neurological Function. Fluids Barriers CNS (2019) 16:3. 10.1186/s12987-019-0123-z 30691500 PMC6350359

[19] Nagatake T Zhao YC Ito T Itoh M Kometani K Furuse M. Selective Expression of Claudin-5 in Thymic Endothelial Cells Regulates the Blood-Thymus Barrier and T-cell Export. Int Immunol (2021) 33:171–82. 10.1093/intimm/dxaa069 PMC7936066 33038259

[20] Bunting MD Comerford I McColl SR. Finding Their Niche: Chemokines Directing Cell Migration in the Thymus. Immunol Cell Biol (2011) 89:185–96. 10.1038/icb.2010.142 21135866

[21] Aili A Zhang J Wu J Wu H Sun X He Q. Ccr2 Signal Facilitates Thymic Egress by Priming Thymocyte Responses to Sphingosine-1-Phosphate. Front Immunol (2018) 9:1263. 10.3389/fimmu.2018.01263 29930553 PMC6001116

[22] Teng F Zhou Y Jin R Chen Y Pei X Liu Y. The Molecular Signature Underlying the Thymic Migration and Maturation of Tcrαβ+ CD4+ CD8 Thymocytes. PloS One (2011) 6:e25567. 10.1371/journal.pone.0025567 22022412 PMC3192722

[23] Yanagida K Hla T. Vascular and Immunobiology of the Circulatory Sphingosine 1-Phosphate Gradient. Annu Rev Physiol (2017) 79:67–91. 10.1146/annurev-physiol-021014-071635 27813829 PMC5500220

[24] Lo Buono N Parrotta R Morone S Bovino P Nacci G Ortolan E. The CD157-integrin Partnership Controls Transendothelial Migration and Adhesion of Human Monocytes. J Biol Chem (2011) 286:18681–91. 10.1074/jbc.M111.227876 PMC3099685 21478153

[25] Love PE Bhandoola A. Signal Integration and Crosstalk During Thymocyte Migration and Emigration. Nat Rev Immunol (2011) 11:469–77. 10.1038/nri2989 PMC3710714 21701522

[26] Lopes N Sergé A Ferrier P Irla M. Thymic Crosstalk Coordinates Medulla Organization and T-Cell Tolerance Induction. Front Immunol (2015) 6:365. 10.3389/fimmu.2015.00365 26257733 PMC4507079

[27] Nitta T Ohigashi I Nakagawa Y Takahama Y. Cytokine Crosstalk for Thymic Medulla Formation. Curr Opin Immunol (2011) 23:190–7. 10.1016/j.coi.2010.12.002 21194915

[28] Zhu M Chin RK Tumanov AV Liu X Fu YX. Lymphotoxin Beta Receptor is Required for the Migration and Selection of Autoreactive T Cells in Thymic Medulla. J Immunol (Baltimore Md: 1950) (2007) 179:8069–75. 10.4049/jimmunol.179.12.8069 18056347

[29] Sitnik KM Wendland K Weishaupt H Uronen-Hansson H White AJ Anderson G. Context-Dependent Development of Lymphoid Stroma From Adult Cd34(+) Adventitial Progenitors. Cell Rep (2016) 14:2375–88. 10.1016/j.celrep.2016.02.033 26947077

[30] Kabashima K Banks TA Ansel KM Lu TT Ware CF Cyster JG. Intrinsic Lymphotoxin-Beta Receptor Requirement for Homeostasis of Lymphoid Tissue Dendritic Cells. Immunity (2005) 22:439–50. 10.1016/j.immuni.2005.02.007 15845449

[31] Wang YG Kim KD Wang J Yu P Fu YX. Stimulating Lymphotoxin Beta Receptor on the Dendritic Cells is Critical for Their Homeostasis and Expansion. J Immunol (Baltimore Md: 1950) (2005) 175:6997–7002. 10.4049/jimmunol.175.10.6997 16272360

[32] Hikosaka Y Nitta T Ohigashi I Yano K Ishimaru N Hayashi Y. The Cytokine RANKL Produced by Positively Selected Thymocytes Fosters Medullary Thymic Epithelial Cells That Express Autoimmune Regulator. Immunity (2008) 29:438–50. 10.1016/j.immuni.2008.06.018 18799150

[33] Hale JS Fink PJ. Back to the Thymus: Peripheral T Cells Come Home. Immunol Cell Biol (2009) 87:58–64. 10.1038/icb.2008.87 19030016 PMC2679673

[34] Hale JS Boursalian TE Turk GL Fink PJ. Thymic Output in Aged Mice. Proc Natl Acad Sci U.S.A. (2006) 103:8447–52. 10.1073/pnas.0601040103 PMC1482512 16717190

