# Supplementary material for: Thymic Egress Is Regulated by T Cell-Derived LTβR Signal and via Distinct Thymic Portal Endothelial Cells
Source: Front Immunol. 2021 Jul 1;12:707404. doi: 10.3389/fimmu.2021.707404 (PMC8281811; doi:10.3389/fimmu.2021.707404)
Supplement: Supplementary file 1 [file DataSheet_1.docx]

***Supplementary Material***


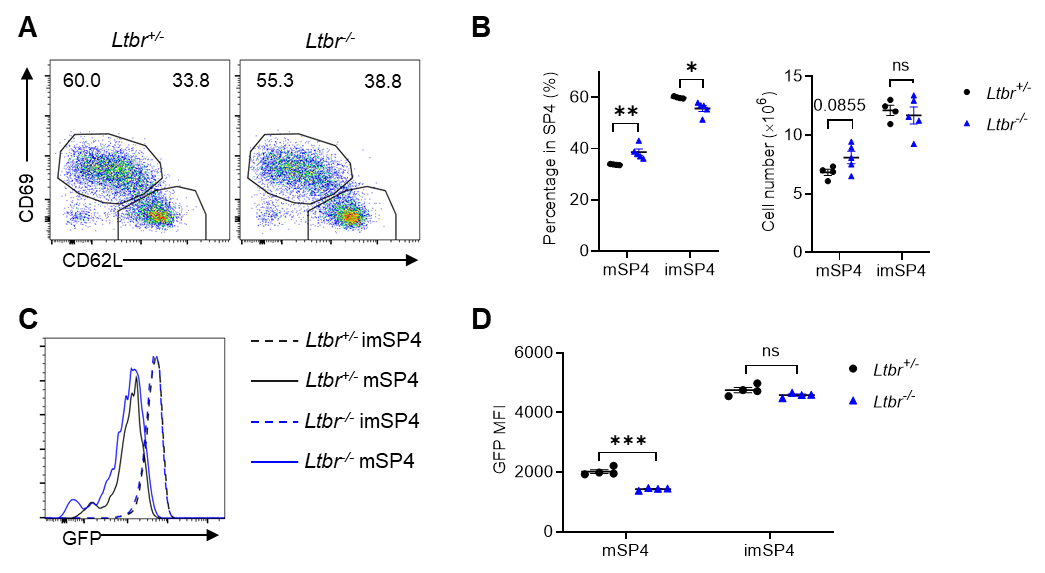


**Supplementary Figure 1. LTβR regulates thymic egress.**

**(A, B)** Flow cytometric analysis of SP4 thymocytes in *Ltbr*^-/-^ and littermate control mice. (A) Immature or mature thymocytes are gated according to CD62L and CD69. Representative dot plots are shown. (B) The graphs display the statistical analysis of the percentage of immature or mature CD4^+^ SP thymocytes among total SP4 population and their numbers. Mean ± SEM; n=4 and 5. Data are representative of at least three independent experiments. **(C, D)** Flow cytometric analysis of GFP expression on SP4 thymocytes in *Ltbr*^-/-^Rag2pGFP and littermate control mice. (C) Representative histogram plots are shown. (D) The graphs display the statistical analysis of the GFP MFI of populations as in **C**. Mean ± SEM; n=4. Data are representative of at least three independent experiments. ns, P>0.05; *, P<0.05; **, P<0.01; ***, P<0.001 (unpaired Student’s *t*-test).


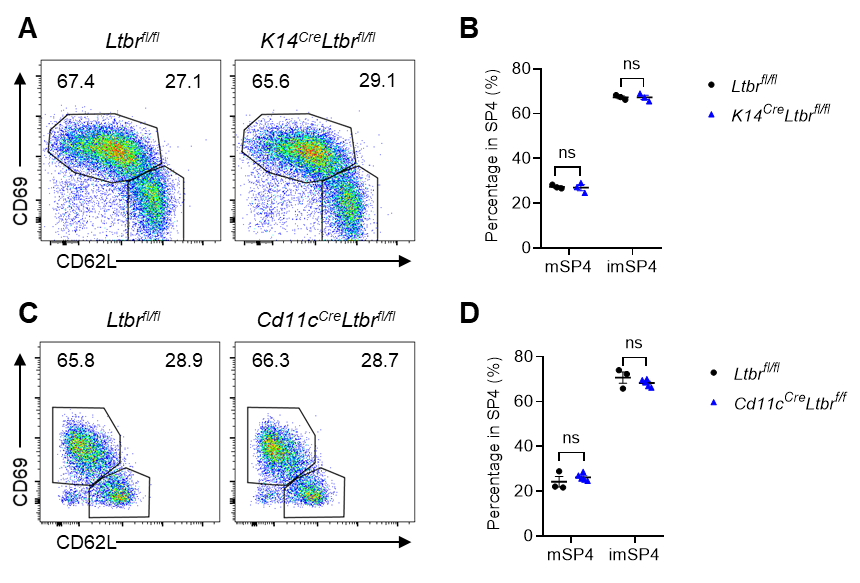


**Supplementary Figure 2. TEC- or DC-derived LTβR doesn’t regulate thymic egress.**

**(A, B)** Flow cytometric analysis of SP4 thymocytes in *K14*^Cre^*Ltbr*^fl/fl^ and littermate control mice. (A) Immature or mature thymocytes are gated according to CD62L and CD69. Representative dot plots are shown. (B) The graphs display the statistical analysis of the percentage of immature or mature CD4^+^ SP thymocytes among total SP4 population. Mean ± SEM; n=3. Data are representative of at least three independent experiments. **(C, D)** Flow cytometric analysis of SP4 thymocytes in *Cd11c*^Cre^*Ltbr*^fl/fl^ and littermate control mice. (C) Immature or mature thymocytes are gated according to CD62L and CD69. Representative dot plots are shown. (D) The graphs display the statistical analysis of the percentage of immature or mature CD4^+^ SP thymocytes among total SP4 population. Mean ± SEM; n=3. Data are representative of two independent experiments. ns, P>0.05 (unpaired Student’s *t*-test).


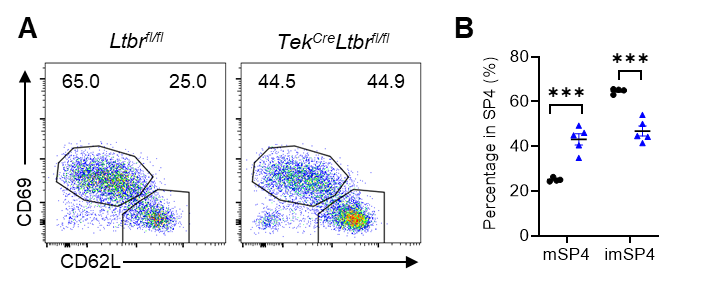


**Supplementary Figure 3. EC-derived LTβR regulates thymic egress.**

**(A, B)** Flow cytometric analysis of SP4 thymocytes in *Tek*^Cre^*Ltbr*^fl/fl^ and littermate control mice. (A) Immature or mature thymocytes are gated according to CD62L and CD69. Representative dot plots are shown. (B) The graphs display the statistical analysis of the percentage of immature or mature CD4^+^ SP thymocytes among total SP4 population. Mean ± SEM; n=4 and 5. Data are representative of at least three independent experiments. ns, P>0.05; *, P<0.05; **, P<0.01; ***, P<0.001 (unpaired Student’s *t*-test).


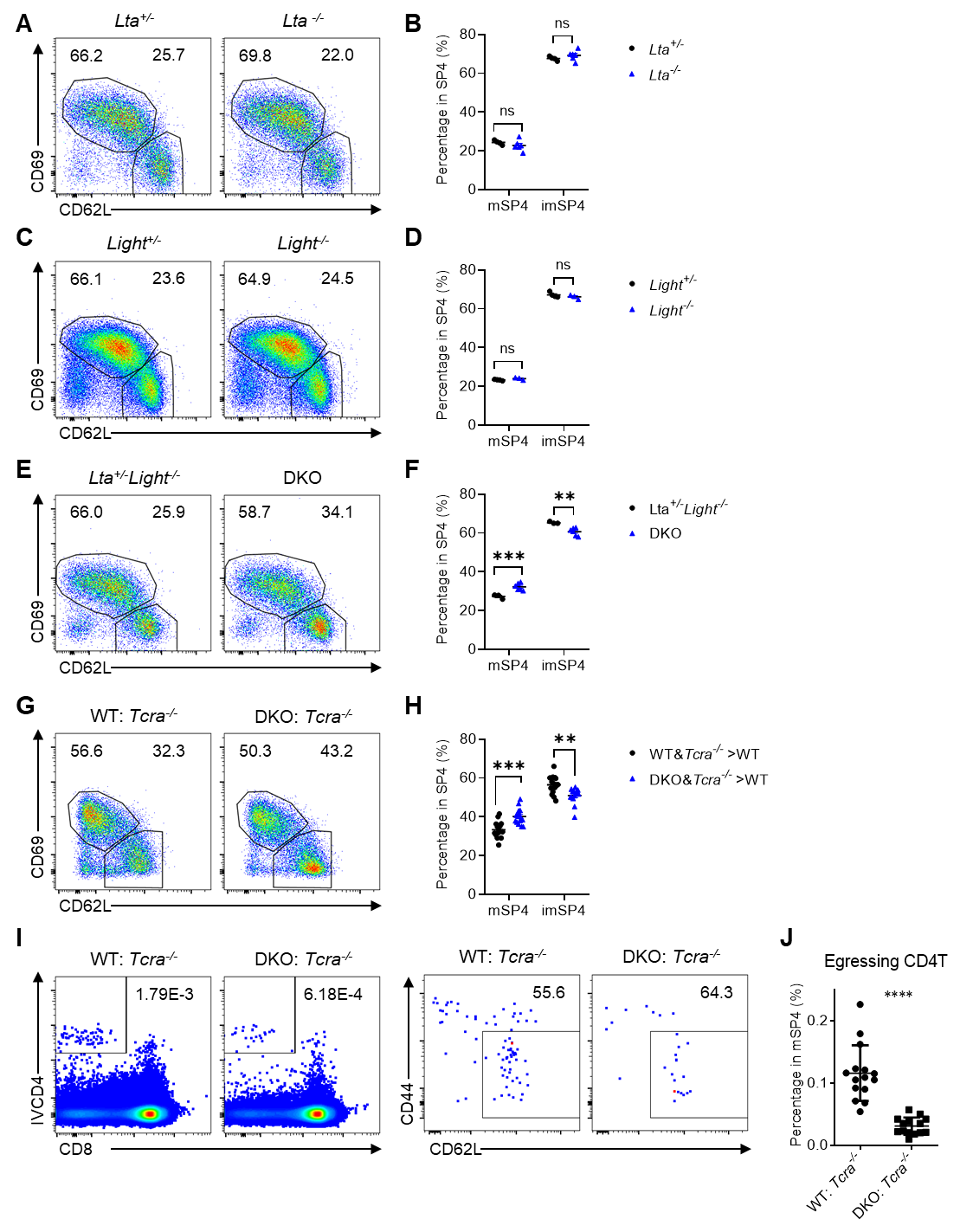


**Supplementary Figure 4. LT and LIGHT expressed on T cells redundantly regulate thymic egress.**

**(A, B)** Flow cytometric analysis of SP4 thymocytes in *Lta*^-/-^ and littermate control mice. (A) Immature or mature thymocytes are gated according to CD62L and CD69. Representative dot plots are shown. (B) The graphs display the statistical analysis of the percentage of immature or mature CD4^+^ SP thymocytes among total SP4 population. Mean ± SEM; n=5. Data are representative of at least three independent experiments. **(C, D)** Flow cytometric analysis of SP4 thymocytes in *Light*^-/-^ and littermate control mice. (C) Immature or mature thymocytes are gated according to CD62L and CD69. Representative dot plots are shown. (D) The graphs display the statistical analysis of the percentage of immature or mature CD4^+^ SP thymocytes among total SP4 population. Mean ± SEM; n=3 and 4. Data are representative of at least three independent experiments. **(E, F)** Flow cytometric analysis of SP4 thymocytes in *Lta*^-/-^*Light*^-/-^ and littermate control mice. (E) Immature or mature thymocytes are gated according to CD62L and CD69. Representative dot plots are shown. (F) The graphs display the statistical analysis of the percentage of immature or mature CD4^+^ SP thymocytes among total SP4 population. Mean ± SEM; n=3 and 6. Data are representative of at least three independent experiments. **(G**-**J)** Flow cytometric analysis of SP4 thymocytes in bone marrow chimeric mice with deficiency of both LTβR ligands in T cells. (G) Immature or mature thymocytes are gated according to CD62L and CD69. Representative dot plots of SP4 thymocytes are shown. (H) The graphs display the statistical analysis of the percentage of immature or mature CD4^+^ SP thymocytes among total SP4 population. Mean ± SEM; n=15. Data are pooled from three independent experiments. (I) Representative dot plots of IVCD4-labeled thymocytes are shown(left). Egressing CD4 T cells are gated from IVCD4-labeled thymocytes (right). (J) The graphs display the statistical analysis of the percentage of egressing CD4 T cells among total mSP4 population. Mean ± SEM; n=15 and 14. Data are pooled from three independent experiments. ns, P>0.05; *, P<0.05; **, P<0.01; ***, P<0.001 (unpaired Student’s *t*-test).


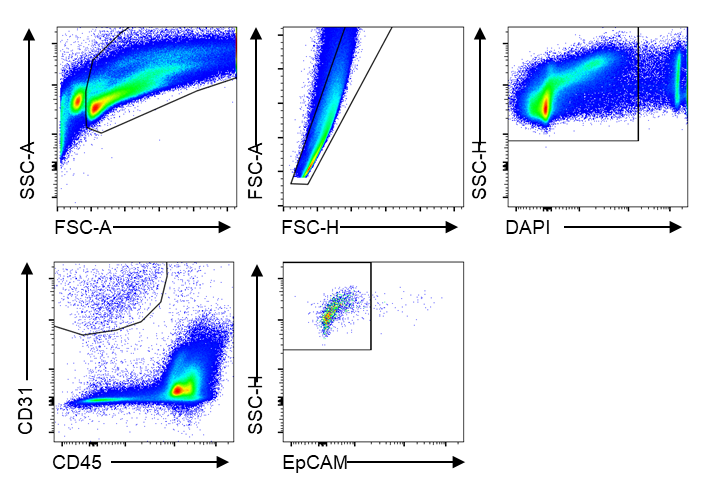


**Supplementary Figure 5. Sorting strategy for thymic ECs.**

Cells collected after enrichment by percoll were sorted for thymic ECs. The dot plots show the gating of non-debris cells, single cells, live cells, ECs and non-epithelial cells, successively. ECs are defined as CD31^+^CD45^-^EpCAM^-^.


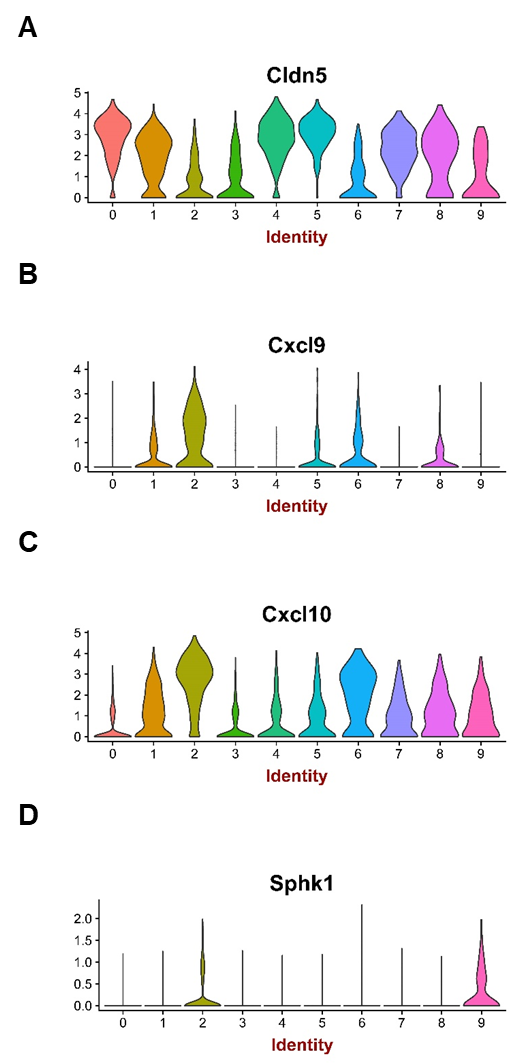


**Supplementary Figure 6. Signature markers of eTPECs.**

**(A)** Violin plot analysis of Cldn5 gene expression on different clusters of thymic ECs.

**(B)** Violin plot analysis of Cxcl9 gene expression on different clusters of thymic ECs.

**(C)** Violin plot analysis of Cxcl10 gene expression on different clusters of thymic ECs.

**(D)** Violin plot analysis of Sphk1 gene expression on different clusters of thymic ECs.
